# Assessing the association between an early and recommended number of focused antenatal care visits and the number of prenatal care content received before delivery in Ethiopia

**DOI:** 10.1371/journal.pone.0282694

**Published:** 2023-03-03

**Authors:** Mary Rachael Kpordoxah, Abdul-Nasir Issah, Daudi Yeboah, Kalayu Brhane Mruts, Michael Boah

**Affiliations:** 1 Department of Global and International Health, School of Public Health, University for Development Studies, Tamale, Ghana; 2 Department of Health Services, Policy, Planning, Management, and Economics, School of Public Health, University for Development Studies, Tamale, Ghana; 3 Department of Epidemiology, Biostatistics, and Disease Control, School of Public Health, University for Development Studies, Tamale, Ghana; 4 Curtin School of Population Health, Curtin University, Perth, Australia; 5 School of Public Health, Debre Berhan University, Debre Berhan, Ethiopia; Menzies School of Health Research, AUSTRALIA

## Abstract

**Background:**

Early and frequent antenatal care (ANC) has been linked to better pregnancy outcomes. This study assessed whether having at least four ANC contacts was associated with increased prenatal care content if the first visit was started in the first trimester in Ethiopia.

**Methods:**

Data from the 2019 Ethiopia Mini Demographic and Health Survey on 2894 women aged 15–49 who received ANC during their last pregnancy were analyzed. The sum of women’s responses to six questions about ANC components (blood pressure taken, urine sample taken, blood sample taken, provided or bought iron tablet, counselling by a health worker on nutrition, and told about pregnancy complications) was used to construct a composite score of routine ANC components. The main predictor was a combination of the timing of the first contact and the number of ANC contacts before birth.

**Results:**

We found that 28.7% of women who began ANC early made at least four ANC contacts. More than one-third (36%) received all six components, with blood pressure monitoring being the most common (90.4%). After adjusting for potential confounding factors, women who had at least four contacts and booked early were substantially more likely than their counterparts to get a factor-of-one increase in the number of components received (IRR = 1.08; 95% CI: 1.03, 1.10).

**Conclusion:**

We found a strong association between increased prenatal care content and early ANC with at least four contacts. However, less than a third of women in the study setting had at least four contacts, with the first occurring in the first trimester. In addition, less than half of women received essential prenatal care interventions before delivery. The findings suggest that the WHO’s new guidelines for ANC frequency and timing may be challenging to implement in some countries, such as Ethiopia, that already have low coverage of four or more contacts. If the recommendations are adopted, effective strategies for increasing early starts and increasing contacts are required.

## Introduction

According to the World Health Organization (WHO), the death of a woman from any pregnancy-related cause or within 42 days of pregnancy, expressed as a ratio to 100,000 live births in a specific population, is termed “maternal mortality” [1]. Globally, there has been a reduction in the number of maternal deaths, although the numbers in some parts of the world, particularly in the least developed countries, are still unacceptably high. In 2017, of the 295,000 maternal deaths that occurred in the world, 196,000 occurred in sub-Saharan Africa (SSA), which was 66% of the global maternal deaths [2]. A maternal death can have a negative impact on families’ physical and mental health, in addition to the tragic loss of life [3]. Children whose mothers have died have a significantly higher mortality rate, according to studies [4–6]. Other documented effects of maternal mortality include catastrophic payments and reduced household income [7,8].

The maternal mortality ratio in Ethiopia has declined from 1030 deaths per 100,000 live births in 2000 to 401 deaths per 100,000 live births in 2017, which is about two times higher than the global average of 211 deaths per 100,000 live births in the same year [2]. The Sustainable Development Goal (SDG) 3.1 aims to reduce the global maternal mortality ratio to less than 70 deaths per 100,000 live births. The risk of maternal deaths can be reduced when women have access to high-quality care before, during, and after childbirth. Antenatal care (ANC) has been shown to be an important global indicator of maternal health and an opportunity for prevention and management of existing and potential causes of maternal and newborn mortality and morbidity [9]. The risk of adverse pregnancy outcomes, such as stillbirths, preterm births, and low birthweight, can be mitigated through preventative measures provided to pregnant women at ANC, such as deworming, iron and folic acid supplementation, and intermittent preventive treatment of malaria during pregnancy (IPTp) [10,11].

Ethiopia is one of the countries that lags behind in terms of coverage of ANC. Less than half (43%) of reproductive women in the country have at least four contacts throughout pregnancy, and slightly over a quarter (28%) of women initiate ANC in the first trimester [12]. In addition, the available literature in the study area is primarily focused on the number of visits made for ANC services, specifically the prevalence and predictors of making at least four ANC contacts [13–17]. Little is known about the proportion of women who book ANC early and are able to make at least four contacts with the healthcare system before delivery, as well as the effect of early ANC initiation with frequent visits on the receipt of essential prenatal services by women in countries with low coverage of early ANC initiation and four or more contacts, such as Ethiopia. During our search for literature, however, we came across a nationwide study by Woldeamanuel and Belachew, who used the 2016 Ethiopia DHS dataset to examine the factors associated with the number of ANC components received from a skilled provider. Women who had at least four ANC visits received more contents than those who only had one visit, according to their findings [18]. Shiferaw and colleagues’ community-based panel study, which examined the extent of components of ANC received and associated factors among Ethiopian women, was also detected [19]. However, they did not establish a link between the number of ANC contacts and the components of care received. We could not find a study that investigated the relationship between starting ANC early with frequent visits and receipt of prenatal care content. As a result, more research is required.

The current study examined the number of components of prenatal care received by women who made at least four ANC contacts, the first of which happened during the first trimester in Ethiopia, using the 2019 Ethiopia Mini Demographic and Health Survey (EMDHS). Specifically, given Ethiopia’s existing fragile health system, we investigated whether increasing content of care received during pregnancy is associated with an early ANC start with at least four ANC contacts. The current study fulfils two functions. First, it determined the fraction of women who begin ANC in the first trimester and are able to make at least four contacts before delivery. Second, it investigated if timely ANC with frequent contacts is associated with an increase in the number of prenatal care interventions obtained by women prior to birth in Ethiopia.

## Materials and methods

### Study design and data source

The analysis in this study is based on data collected from the 2019 EMDHS, a nationally representative cross-sectional household survey. The sampling frame used for the 2019 EMDHS is a frame of all census enumeration areas (EAs) created by the Central Statistical Agency (CSA).

The 2019 EMDHS sample was stratified and selected in two stages. Each region was stratified into urban and rural areas, yielding 21 sampling strata. In the first stage, 305 EAs were selected with a probability proportional to EA size and with independent selection in each sampling stratum. In the second stage of selection, a fixed number of 30 households per EA were selected with an equal probability of systematic selection from the newly created household listing. The sampling details for the 2019 EMDHS, data collection methods and tools, as well as quality control measures, have been documented in the full report [12].

### Sample

This study utilised the individual recode (women’s file) dataset for analysis. A total of 2894 women aged 15–49 years who had received ANC services during their most recent pregnancy were analyzed. This sample was weighted to account for the complex study design used by the DHS program in its surveys.

### Variables

#### Outcome variable

The outcome variable in this study is the number of components of prenatal care received by women of reproductive age during their most recent pregnancy. We developed composite scores of routine components of ANC based on the sum of women’s responses to a set of six questions regarding the component of prenatal care they received, including: 1) blood pressure taken; 2) urine sample taken; 3) blood sample taken; 4) given or bought iron tablet; 5) counselling by a health worker about nutrition; and 6) told about the signs of pregnancy complications. For each of these questions, the response options were yes (score = 1) or no (score = 0). The total scores ranged from 0 to 6, with “0” implying that none of the components was received and “6” implying that all six investigated components were received. We would like to point out that the six components studied in this study are not all-inclusive. Weight measurement, deworming, and birth preparation discussions are also recommended as part of the standard ANC guidelines for all pregnant women in Ethiopia [19]. However, these data were not available in the dataset that we used for the current study.

### Exposure variable

The exposure variable investigated in this study is a combination of the timing of the first ANC contact and the total number of contacts made before delivery, labelled “early ANC with at least four ANC contacts”. The exposure variable was dichotomized into “No” and Yes” for analysis purposes. Women who started ANC early and had at least four contacts before delivery were classified as “Yes” (for early ANC with at least four ANC contacts). Those who started early but had fewer than four contacts were classified as “No”. Those who started late and made fewer than or more than four contacts were likewise classified as “No”. In the current study, a pregnant woman is defined as having an early ANC start when she reports that her first ANC contact was initiated during the first three months of her most recent pregnancy.

### Covariates

We included the following demographic, obstetric, and socioeconomic characteristics as covariates: maternal age, educational level, current marital status, number of children ever born, region, type of place of residence, and wealth index based on previous studies conducted [19,20]. Most of these variables were used as they existed in the dataset. Using the existing DHS variables, however, new variables such as respondent age group, number of children ever born, and educational level were created. It is worth noting that the covariates included in the current study are not the only known factors that can influence women’s use of health services, as reported in the literature; cultural beliefs and perceptions about pregnancy, ease of access to health facilities, cost of services, and ANC quality have all been identified as factors influencing women’s use of health services, particularly ANC services in low-resource settings [21–23]. However, due to a lack of availability of these factors in the dataset, we were unable to include them in our analysis.

### Statistical analysis

Descriptive statistics were computed for the background characteristics and both for each question on the routine components of ANC received and the distribution of the components. We performed unadjusted and adjusted multivariable Poisson regressions to examine the association between the exposure variable and the outcome variable. Multicollinearity between variables was checked using the Variance Inflation Factor (VIF) method before building the adjusted model. The mean VIF was 1.49 (range: 1.01–1.94). To adjust for the complex survey design used in the DHS, sampling weight was applied in all the analyses [24]. All of the statistical analyses were conducted using Stata version 13.0 (StataCorp. LP, College Station, USA). The statistical significance was set at a p<0.05.

### Ethical consideration

This study focused on the analysis of secondary data from the Ethiopian Mini Demographic and Health Survey 2019. MEASURE DHS/Inner City Fund (ICF) International gave the authors permission to use the dataset. The DHS Program adheres to industry guidelines for protecting the privacy of respondents. ICF International guarantees that the survey complies with the Human Subjects Protection Act of the United States Department of Health and Human Services. Before the survey, the DHS project sought and received the required ethical approval. Informed consent was obtained from participants. Parents or guardians of respondents younger than 18 years old provided written informed consent. More information on data and ethical principles can be found online at the DHS program website (https://dhsprogram.com/methodology/Protecting-the-Privacy-of-DHS-Survey-Respondents.cfm). This study, therefore, did not require any additional approvals.

## Results

### Background characteristics of the women analysed in this study

The obstetric, demographic, and economic characteristics of the women studied are presented in Table 1. Of the women investigated, 1680 (58.1%) had at least four contacts, of which 832 (28.7%) made the first contact in the first trimester. Approximately 44.0% of women received no formal education, and 33.6% were from households below the middle class in terms of wealth. The majority of women were in the age group of 25–34 years (53.2%), married (93.5%), and resided in rural areas (70.2%).

**Table 1:** Background characteristics of the women analysed (N = 2,894).

Variable	Frequency	Weighted %
Number of antenatal care contacts		
1–3	1214	40.9
4 or more	1680	58.1
**Early ANC with at least four contacts**		
No	2062	71.3
Yes	832	28.7
**Respondent’s age group (years)**		
15–24	750	25.9
25–34	1539	53.2
35–49	605	20.9
**Educational level**		
No formal education	1266	43.7
Primary	1147	39.6
At least secondary education	481	16.6
**Current marital status**		
Not in union	168	5.8
In a union	2726	94.2
**Number of children ever born**		
1	685	23.7
2	611	21.1
3	421	14.5
4 or more	1177	40.7
**Region**		
Tigray	270	9.3
Afar	32	1.1
Amhara	697	24.1
Oromia	1072	37.0
Somali	63	2.2
Benishangul	39	1.3
SNNPR	558	19.3
Gambela	16	0.6
Harari	9	0.3
Addis Ababa	121	4.2
Dire Dawa	17	0.6
**Type of place of residence**		
Urban	861	29.8
Rural	2033	70.2
**Wealth index**		
Poorest	398	13.8
Poorer	576	19.9
Middle	586	20.2
Richer	573	19.8
Richest	760	26.3

ANC: Antenatal care; SNNPR: South Nation, Nationalities, and People Region.

### Distribution of the prenatal care components received by women before delivery

The distribution of the prenatal components received by the women is presented in Table 2. The results indicated that receipt of the six routine components investigated varied greatly; blood pressure measurement was the most commonly reported component (88.2%), and information on pregnancy complications was the least (60.5%). Overall, 2.2% of the women did not receive any of the routine interventions, 61.8% received between 1 and 5 components, and 36.0% (95% CI: 32.2, 39.9) of the women received all six routine components of ANC.

**Table 2:** Distribution of the components of prenatal care received by women before delivery in this study (N = 2,894).

Responses to questions on antenatal care interventions	Frequency	Weighted %
Blood pressure taken		
No	341	11.8
Yes	2553	88.2
**Urine sample taken**		
No	752	26.0
Yes	2142	74.0
**Blood sample taken**		
No	608	21.0
Yes	2286	79.0
**Given or bought iron tablet /syrup**		
No	663	22.9
Yes	2231	77.1
**Counselling by a health worker about nutrition**		
No	831	28.7
Yes	2063	71.3
**Told about the signs of pregnancy complications**		
No	1143	39.5
Yes	1751	60.5
**Total number of antenatal care components received**		
0	65	2.2
1	144	5.0
2	193	6.7
3	255	8.8
4	512	17.7
5	684	23.6
6	1041	36.0

### Association between early antenatal care with at least four contacts and the number of prenatal care components received before delivery

The results of Poisson regression analyses of the association between an early ANC with at least four contacts are presented in Table 3. The results indicate that early ANC with at least four contacts before delivery was significantly associated with the number of prenatal care components received by women before delivery. The number of components received increased among women who started ANC early and made at least four contacts compared to their counterparts. The association remained after controlling for potential confounding factors. In the adjusted model, women who had at least four contacts and booked early were significantly more likely than their counterparts to have the number of components received increase by a factor of one (IRR = 1.08; 95% CI: 1.03, 1.13; *p*< 0.01). Among the covariates, factors, including maternal educational level, region, and wealth index, were associated with the number of components of care received. Women with at least a secondary level of education were more likely to receive more components than those with no formal education (IRR = 1.13; 95% CI: 1.06, 1.21; *p*<0.001). Women in Oromia (IRR = 0.86; 95% CI: 0.79, 0.94; *p* < 0.01), Harari (IRR = 0.93; 95% CI: 0.87, 0.99; *p <* 0.05), and Dire Dawa (IRR = 0.93; 95% CI: 0.88, 0.99; *p*< 0.05) were more likely to receive fewer components than women in Tigray. Women from the middle, richer, and richest households had one more ANC component than women from the poorest households.

**Table 3:** Association between early antenatal care with at least four contacts and the number of prenatal care components received before delivery (N = 2,894).

Variable	Model 1	Model 2
	IRR [95% CI]	IRR [95% CI]
Early ANC with at least four contacts		
No	1.00	
Yes	1.16***[1.11,1.22]	1.08**[1.03,1.13]
**Respondent’s age group (years)**		
15–24		
25–34		0.99[0.94,1.04]
35–49		0.96[0.90,1.03]
**Educational level**		
No formal education		
Primary		1.05[1.00,1.10]
At least secondary education		1.13***[1.06,1.21]
**Current Marital Status**		
Not in union		1.00
In a union		0.99[0.93,1.06]
**Number of children ever born**		
1		1.00
2		1.01[0.96,1.06]
3		1.03[0.96,1.10]
4 or more		1.02[0.95,1.10]
**Region**		
Tigray		1.00
Afar		1.04[0.95,1.12]
Amhara		1.05[0.98,1.12]
Oromia		0.86**[0.79,0.94]
Somali		0.91[0.80,1.03]
Benishangul		0.92[0.85,1.00]
SNNPR		0.92[0.84,1.01]
Gambela		0.96[0.88,1.05]
Harari		0.93*[0.87,0.99]
Addis Ababa		0.99[0.93,1.06]
Dire Dawa		0.93*[0.88,0.99]
**Type of place of residence**		
Urban		
Rural		1.01[0.95,1.08]
**Wealth index**		
Poorest		
Poorer		1.05[0.97,1.13]
Middle		1.15**[1.05,1.25]
Richer		1.16**[1.05,1.28]
Richest		1.25***[1.14,1.37]

Exponentiated coefficients; 95% confidence intervals in brackets.

* *p* < 0.05

** *p* < 0.01

*** *p* < 0.001.

ANC: Antenatal care; IRR: Incidence rate ratio; SNNPR: South Nation, Nationalities and People Region.

## Discussion

The current study assessed whether having at least four ANC contacts was associated with more prenatal care content if the first visit started in the first trimester in a country with low ANC initiation and contact coverage. It has been acknowledged that women in low- and middle-income countries with timely ANC initiation have a higher likelihood of achieving four or eight contacts [25,26]. In fact, every one-month delay in initiating ANC reduces the total number of contacts that must be made prior to delivery by approximately one [26]. According to the findings of the current study, less than a third (28.7%) of women who initiated ANC early had four or more contacts before birth. A nationwide report from the study setting shows that less than half of reproductive women in the country have at least four contacts throughout pregnancy, and slightly over a quarter of women initiate ANC in the first trimester in 2019 [12]. However, because contact time and frequency are reported independently, we are unable to compare the current study’s findings with the previous literature in the study setting. Nonetheless, our finding falls in the range of approximately 15% to 89% reported by Benova and her colleagues when they assessed ANC coverage in LMICs [27]. Our rate is also slightly higher than the 25% found by a study that examined the factors that influence ANC and skilled birth attendance in 34 SSA countries [28].

Our results also indicated that the coverage of the six routine prenatal components investigated varied greatly. The data showed that approximately 2% of women did not receive any of the components, whereas 36% received all six components, with blood pressure measurement being the most frequently reported component received. This finding is consistent with global trends, which show that blood pressure measurement is the most commonly provided ANC component [29]. Information on pregnancy complications was the least common of the six components, with less than two-thirds of the women surveyed receiving it, which is comparable to the prevalence in other LMICs [27]. Nevertheless, this finding is cause for concern given that complications during pregnancy and delivery cause maternal and infant mortality. Poor counselling and provider-client interaction are issues that public health institutions in Ethiopia face, and these factors predict client satisfaction with prenatal care services [30,31]. As a result, increased efforts are needed to ensure that all pregnant women receive information on pregnancy-related complications, allowing for early detection of warning signs and prompt action to reduce the risk of pregnancy-related adverse health outcomes.

Consistent with previous research [27], the current study found a statistically significant relationship between the number of prenatal care components received prior to delivery and the number of ANC contacts made during pregnancy. Specifically, we found that having at least four ANC contacts, if the first contact occurred in the first trimester increased the number of prenatal care components received by approximately one. In the 2019 EMDHS, women were asked if they had received any of the interventions at least once during their most recent pregnancy. As a result, it is reasonable to say that more visits would result in the woman receiving a greater number of ANC interventions at least once before delivery.

Our findings have policy implications for the study setting. The World Health Organization (WHO) in 2016 recommended at least eight ANC contacts during pregnancy, the first of which should occur in the first trimester, to improve perinatal outcomes and women’s experiences of care [32]. Despite the fact that the current study did not examine compliance with the new recommendations, given the low coverage of four or more contacts among early ANC registrants, we anticipate an even lower coverage of eight or more contacts. Jiwani et al. found that the coverage of eight or more contacts in countries with 50–75% coverage in four or more visits, such as Ethiopia, is approximately 6% [26]. Consequently, we can infer that the new recommendations regarding the time and frequency of ANC contacts may not be “one-size-fits-all,” and country circumstances must be taken into account. Implementing these recommendations in settings with low coverage of four visits and early initiation, such as Ethiopia, will be challenging and require increased efforts and strategies to promote frequent contacts with the health care system throughout pregnancy. Specifically, it will be necessary to address the underlying drivers of low utilisation of ANC services in the country, such as poor access to services, cultural beliefs and perceptions about pregnancy, economic barriers, poor quality of ANC, a lack of decision-making power by women, and a lack of knowledge, especially regarding the correct timing of the first contact [14,17,33–37].

### Strengths and limitations of the current study

The current study’s main strength is that we used a nationally representative dataset. As a result, the findings can be applied to women of reproductive age in the study setting who have used ANC at least once before delivery. Nonetheless, we would like to acknowledge that the six components investigated in this study are not exhaustive. Other interventions, such as weight measurement, deworming, and discussions about birth preparation, are recommended as part of the standard ANC guidelines for all pregnant women in Ethiopia [19]. However, these data were not included in the dataset that we used for the current study. In addition, the covariates used in the current analyses did not include all of the factors known to influence women’s receipt of care during ANC, but only those that were available in the dataset. Another drawback of the current study is that all of the measures used in the analysis, such as the timing, number, and prenatal care components received, were dependent on mothers’ recollections of events during the prenatal period and may be subject to recall bias. The DHS also used a cross-sectional study design, which makes it impossible to draw causal linkages from the results. Finally, women who did not use ANC during their most recent pregnancy were excluded from the current study; the findings can only be applied to women of reproductive age in Ethiopia who have used ANC services at least once during pregnancy.

### Conclusions

We found a strong association between increased prenatal care content and having at least four contacts if ANC starts early. However, less than a third of women in the study setting had at least four contacts, with the first occurring in the first trimester. In addition, less than half of women received essential prenatal care interventions before delivery, and a significant proportion did not receive any intervention despite using ANC. Healthcare providers must make sure that all pregnant women who use the healthcare system get the ANC interventions they need to have a healthy pregnancy. The findings also suggest that the WHO’s new guidelines for ANC frequency and timing may be challenging to implement in some countries, such as Ethiopia, which already has low coverage of four or more contacts. If the recommendations are adopted, effective strategies for increasing early starts and increasing contacts will be required. In northwest Ethiopia, community-level demand creation and dropout tracing mechanisms were implemented, significantly increasing the frequency of ANC contacts among pregnant women [38]. These strategies can be scaled up across the country.

## Abbreviations

ANC: Antenatal care
AOR: Adjusted odds ratio
DHS: Demographic and Health Survey
IRB: Institutional Review Board
IRR: Incidence Rate Ratio
LMICs: Low- and Middle-Income Countries
SSA: sub-Saharan Africa
SDGs: Sustainable Development Goals
WHO: World Health Organization
